# High Performance of SARS-Cov-2N Protein Antigen Chemiluminescence Immunoassay as Frontline Testing for Acute Phase COVID-19 Diagnosis: A Retrospective Cohort Study

**DOI:** 10.3389/fmed.2021.676560

**Published:** 2021-07-14

**Authors:** Qiaoling Deng, Guangming Ye, Yunbao Pan, Wen Xie, Gui Yang, Zhiqiang Li, Yirong Li

**Affiliations:** ^1^Department of Laboratory Medicine, Zhongnan Hospital of Wuhan University, Wuhan University, Wuhan, China; ^2^Department of Neurosurgery, Zhongnan Hospital, Wuhan University, Wuhan, China

**Keywords:** COVID-19, SARS-CoV-2N protein antigen, chemiluminescence immunoassay, testing strategies, diagnostic performances

## Abstract

**Objectives:** COVID-19 emerged and rapidly spread throughout the world. Testing strategies focussing on patients with COVID-19 require assays that are high-throughput, low-risk of infection, and with small sample volumes. Antigen surveillance can be used to identify exposure to pathogens and measure acute infections.

**Methods:** A total of 914 serum samples, collected from 309 currently infected COVID-19 patients, 48 recovered ones, and 410 non-COVID-19 patients, were used to measure N protein antigen levels by a chemilumineseent immunoassay. Diagnostic performances were analyzed in different periods after onset.

**Results:** There was a high level of N protein antigen in COVID-19 patients (0.56 COI), comparing to the recovered patients (0.12 COI) and controls (0.19 COI). In receiver-operating characteristic curve analysis, the area under the curve of serum N protein antigen was 0.911 in the first week after onset. In this period, Sensitivity and specificity of serologic *N* protein antigen testing was 76.27 and 98.78%. Diagnosis performance of specific antibodies became better from the third week after onset. Subgroup analysis suggested that severe patients had higher levels of antigens than mild patients.

**Conclusions:** High level of serum antigen suggested early infection and serious illness. Serum N protein antigen testing by chemiluminescence immunoassay is considered as a viable assay used to improve diagnostic sensitivity for current patients.

## Introduction

Coronavirus disease 2019 (COVID-19), caused by the severe acute respiratory syndrome coronavirus 2 (SARS-CoV-2), was first reported in Wuhan at the end of 2019 (1). Although early cases were reported to link to a large seafood market, later patients confirmed obvious person-to-person transmission of the disease. The COVID-19 pandemic has now spread globally and spawned a global public health crisis. As of early April 2021, more than 146,054,170 people have been diagnosed and more than 3,092,000 have been killed (2).

Molecular assay was thought to be the gold standard for COVID-19 confirmation, but a lot of studies showed that COVID-19 patients may have initial reverse transcriptase polymerase chain reaction (RT-PCR) negative results (3, 4). The false negative results occurred frequently although reasons for false negatives of the molecular assay were found to relate to disease course, having insufficient cellular material for detection, and improper extraction of nucleic acid from clinical materials (3–7).

Serologic antibody assays for the SARS-CoV-2 infection may be used to support the clinical assessment of COVID-19 patients who present late (8, 9). Dynamics surveillance in our previous study showed that the median time for the appearance of SARS-CoV-2 specific IgM antibodies in serum was 9 days after illness onset, whereas the production time of SARS-CoV-2 specific IgG was in the range of day 9 to 12 after the onset of COVID-19 (10). Supporting this view, subsequent study found that the sensitivity of immunochromatographic assay with IgM and IgG combinatorial detection in nucleic acid confirmed cases were 11.1 and 92.9% at the early stage (1–7 days after onset) and intermediate stage (8–14 days after onset), respectively (11). In addition, another study showed that 53 serum samples from COVID-19 patients were found negative for both IgM and IgG, possibly due to the samples being collected at the early stage of illness (12). Therefore, serologic antibody assays for SARS-CoV-2 infection cannot detect acutely infected but previously infected persons, which was generally regarded as an important tool for surveillance and epidemiologic studies. A previous epidemiologic study carried out in Wuhan showed that 0.53% (324/62437) and 1.95% (1200/62437) individuals were positive for the SARS-CoV-2 specific IgM and IgG, respectively, and 0.08% (54/62437) were positive for both antibodies, indicating that the percentage of SARS-CoV-2 specific antibody in Wuhan was low and most Wuhan residents are still susceptible to the SARS-CoV-2 (11).

Like the nucleic acid test for SARS-CoV-2, the specific antigen test is another direct viral detection method, and generally checks samples from nasal, throat swabs, and other upper respiratory tract samples to determine whether an infection with SARS-CoV-2 is present. A meta-analysis based on eight evaluations from five studies showed that the average sensitivity and specificity were 56.2% (95% CI 29.5–79.8%) and 99.5% (95% CI 98.1–99.9%), respectively, indicating that the sensitivity varied considerably across studies (13). Of the five studies, two only used a nasopharyngeal swab, two used both nasopharyngeal and oropharyngeal swabs, and one used mixed samples including bronchoalveolar lavage, nasopharyngeal swabs, and nasopharyngeal aspirate. Similar sensitivity and specificity were found among different sample types, but a large difference was revealed in sensitivity in the high viral load group for antigen tests compared to low viral load.

Although these antigen and nucleic acid assays are useful for the identification of SARS-CoV-2 infection, the process of swabs sampling, specimen pre-treatment, and extraction increase the risk of exposure to viral droplets. In addition, detection of low-throughput is not suitable for large scale screening. However, Venous blood is more easily collected than nasopharyngeal and oropharyngeal swabs. Moreover, the SARS-CoV-2 RNA found in about 40% of COVID-19 patients was associated with organ damage and severe illness. RNA and the nucleocapsid (N) protein are important components of the SARS-CoV-2, we suspected that the N protein was present in the serum of COVID-19 patients in the early stage of illness onset (14, 15). Therefore, we developed the a chemiluminescence immunoassay (CLIA) assay for determining the N protein levels in the serum of COVID-19 patients. It was found that the SARS-CoV-2 antigenemia is very high in the first 2 weeks after symptoms onset, supporting that it is another useful test for the identification of the SARS-CoV-2 infection.

## Materials and Methods

### Patients, Samples, and Data Collection

Consecutive COVID-19 patients who were presented or admitted to Zhongnan Hospital, Wuhan University from January 28 to March 10, 2020, were included to test SARS-COV-2 N protein antigen and specific antibody in serum. The COVID-19 patients and recovered ones enrolled in this study were diagnosed according to diagnostic guidance released by the National Health Commission (16). Healthy volunteers and other virus infected patients who were examined in or admitted to Zhongnan Hospital, Wuhan University from November 18, 2020 to December 6, 2020, were enrolled into the control group. Exclusion criteria of the controls were as follows: (a) positive SARS-COV-2 RNA in throat swabs, (b) patients deficient in basic clinical data. All throat swabs and venous blood samples were collected and processed in zhongnan Hospital, Wuhan University. The remaining sera were collected and stored at −80°C for the test of SARS-CoV-2 specific antibody and N protein antigen. Clinical characteristics, laboratory findings and outcomes were collected from electronic medical records. The study was reviewed and approved by the Ethics Committee of Zhongnan Hospital, Wuhan University. All study objectives have signed an informed consent.

### Real-Time RT-PCR Assay for SARS-CoV-2 RNA

Throat swabs were collected from COVID-19 patients for the testing of SARS-CoV-2 RNA. First, total RNAs were extracted from swab within 3 h using respiratory sample RNA isolation kit (Zhongzhi, Wuhan, China). In brief, 40 μL of cell lysis solution was transferred to a collection tube consisting of the swab followed by vortex for 30 s. After incubation at room temperature for 15 min, the collection tube was centrifugated at 1,000 rpm/min for 5 min. The suspension was used as the template for amplifying by using real-time reverse transcriptase–polymerase chain reaction (RT-PCR) assay kits (Daan Gene, Guangzhou, China). Two target genes, the nucleocapsid (N) protein and the open reading frame 1ab (ORF1ab) of SARS-CoV-2, were simultaneously amplified and detected during the real-time RT-PCR assay. The volume of real-time RT-PCR reaction system was 25 μL, including 2 μL template, 3 μL pure water, 17 μL mixture A and 3 μL mixture B. Each amplification was performed in an Eppendorf tube by ABI prism 7500 (Thermo Fisher Scientific, Waltham, MA, USA). The reaction conditions were as follows: transcription at 50°C for 15 min and pre-denaturation at 95°C for 15 min, following by 45 cycles of denaturation at 94°C for 15 s and extension at 55°C for 45 s. Fluorescence was collected at regular intervals during each extension phase. The lowest detection limit of the real-time RT-PCR assay for two target genes was 500 copies/mL. According to the manufacturer's recommendation, a cycle threshold (Ct) value of <40 was defined as positive.

### Chemiluminescence Immunoassay for Testing SARS-CoV-2 Specific Antibody

Serum IgM antibody against the SARS-CoV-2 N and spike protein were determined by iFlash immunoassay analyzer (Shenzhen Yhlo Biotech Co., Ltd, Shenzhen, China) and iFlash- SARS-CoV-2 IgM detection kit (Shenzhen Yhlo Biotech Co., Ltd, Shenzhen, China) approved by the Chinese Food and Drug Administration (cFDA). It was an indirect two-step immunoassay. Firstly, SARS-CoV-2 specific IgM in the serum binds to the SARS-CoV-2 N and spike protein coated on paramagnetic microparticles to form a complex, after washing away the unbound materimals in the magnetic, acridinium-labeled anti-human IgM conjugate was added for further reaction to form a new complex, then washing again, the Pre-Trigger and Trigger solutions were added to the reaction mixture. Finally, serum SARS-CoV-2 specific IgM levels were calculated based on the resulting relative light units (RLUs) from the reaction mixture via a 2-point calibration curve, and a value of >10 AU/ml was considered to be reactive.

Serum IgG antibody against the SARS-CoV-2 N and spike protein were determined by iFlash-SARS-CoV-2 IgG detection kit (Shenzhen Yhlo Biotech Co., Ltd, Shenzhen, China) approved by the Chinese Food and Drug Administration(cFDA). The principle and procedure for IgG was similar to IgM. The cut off value given by the manufacturer is 10 AU/ml.

### Chemiluminescence Immunoassay for Testing Serum SARS-CoV-2 N Antigen

Serum SARS-CoV-2 N protein antigen was determined using a double antibody sandwich chemiluminescence immunoassay by iFlash immunoassay analyzer (Shenzhen Yhlo Biotech Co., Ltd, Shenzhen, China). Paramagnetic carboxylated-microparticles (Thermo Scientific) were coated with one of 10 candidate specific antibodies (Shenzhen YHLO Biotech Co., Ltd, Shenzhen, China) through cross-linking by N-ethyl-N'-(3-dimethylaminopropyl) carbodiimide (Thermo Scientific) for the N protein antigen capture as previously described. Another antibody was conjugated with NSP-DMAE-NHS (Maxchemtech) for antigen detection. The recombination SARS-CoV-2 nucleocapsid protein (Shenzhen YHLO Biotech) dissolved in healthy human serum was used as the calibrators. Tests can be running after calibration. In the testing, paramagnetic carboxylated-microparticles coated with the capture antibody gathered N protein antigens. After washing the unbound material away, the antibody- N protein antigen -capture antibody compounds reacted with acridinium-labeled antibody. The mixture was retained in a tube under the magnetic field. And then pre-trigger and trigger solution were added to calculate the N protein antigen based on the resulting relative light units (RLUs) via a 2-point calibration curve.

### Statistical Analysis

Statistical analyses were conducted with IBM SPSS version 23.0 software. Normal distribution continuous data were presented in mean ± standard deviation (SD), and skewed distribution continuous data were presented in median and range. Students' test or non-parametric test was used for comparison of continuous data. Chi-square test was used to analyze categorical data. Diagnostic sensitivity and specificity were calculated by the receiver operating characteristic (ROC) curve and area under the curve (AUC). *p* ≤ 0.05 was considered to be statistically significant.

## Results

### Clinical Features of Individuals

There were 504 serum samples from 338 COVID-19 patients. All currently infected and recovered COVID-19 patients had a positive result of the SARS-CoV-2 RNA in the throat swab at least one time. Detailed clinical features and laboratory findings are summarized in Table 1. Time of symptoms onset of COVID-19 patients ranged from 1 to 88 days. Controls consisted of 410 individuals, among which 168 patients were infected with other viruses such as influenza virus (*n* = 73), hepatitis B virus (*n* = 72) and human immunodeficiency virus (*n* = 23), Epstein-Barr virus (*n* = 29), Cytomegalovirus (*N* = 23), and 190 were healthy people.

**Table 1:** Demographics, baseline characteristics of 338 cases.

	**COVID-19 (*****n*** **=** **338)**	
	**Hospitalized**	**Recovered**
Total number of samples/cases	447/309	57/48
Female	156	24
Male	153	24
Mean age	56.04	41.37
**Symptoms**		
Fever	55.62% (*n* = 118)	
Cough	27.81% (*n* = 94)	
Fatigue or muscles ache	10.95% (*n* = 37)	
Chest distress or dyspneic	4.44% (*n* = 15)	
Diarrhea or vomiting	3.25% (*n* = 11)	
Throat pain(sore)	2.07% (*n* = 7)	
Headache or dizzy	2.66% (*n* = 9)	
Running nose	0.89% (*n* = 3)	
Appetite debility	0.89% (*n* = 3)	
Without symptoms	0.30% (*n* = 1)	
NA	25.74% (*n* = 87)	
**Clinical type**		
Mild	49.70% (*n* = 168)	
Severe	26.04% (*n* = 88)	
NA	24.26% (*n* = 82)	
RT-PCR positive (swab)	205	
**Time of onset (serum)**		
<7 days (weeks 1)	118	
7–14 days (weeks 2)	104	
14–21 days (weeks 3)	76	
21–28 days (weeks 4)	56	
>28 days (weeks > 4)	143	
without symptoms	2	
NA	5	

*RT-PCR, reverse transcription-polymerase chain reaction. NA, not available*.

### Lowest Analytical Sensitivity of the SARS-CoV-2 N Protein Chemiluminescence Immunoassay

To establish a highly sensitive SARS-CoV-2 N protein detection system based on chemiluminescence immunoassay, a total of 10 specific antibodies (Supplementary Table 1) against the N- or C-terminal region of SARS-CoV-2 N protein were employed. According to the difference of the capture and detection antibodies, 34 matches were tested. The lowest analytical sensitivity was shown in Supplementary Table 2. A couple of antibodies, Ab02 and Ab04 with lowest analyte concentration of 0.43 pg/ml, were chosen to serve as capture antibody and the detection antibody in further chemiluminescence immunoassay.

### Serum SARS-CoV-2 N Protein Antigen

**Figure 1:**
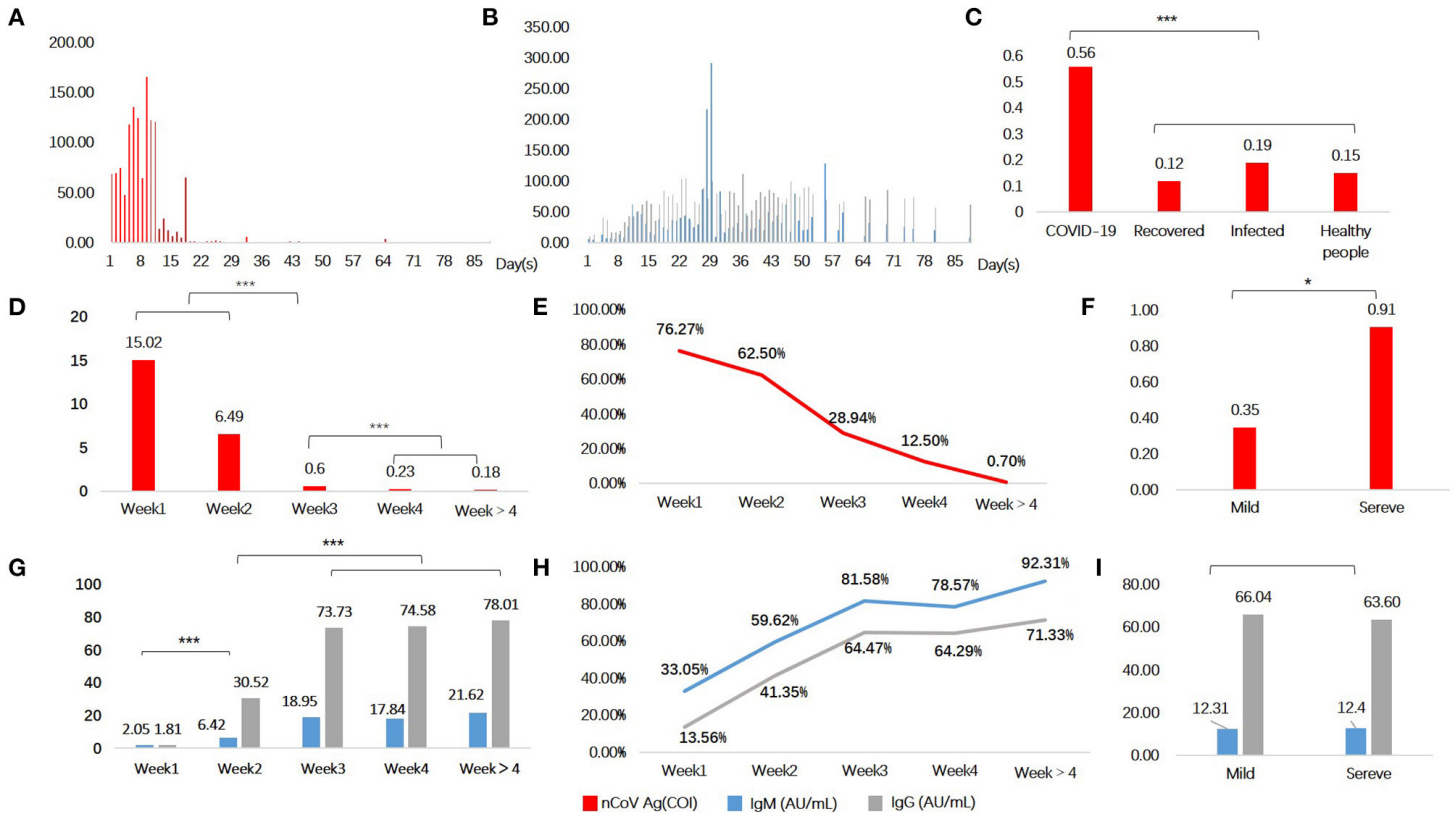
Serological testing in COVID-19 patients and controls. Serum N protein antigen **(A)** and specific antibodies **(B)** as a function of time since symptoms onset. Antigen **(C,D,F)** and antibodies **(G,I)** levels in different groups. Positive rate of serum antigen **(E)** and antibodies **(H)**. Red denotes antigen, blue and grey denote specific antibodies IgM and IgG, respectively. *P < 0.05, ***P < 0.001, and lines without *mean no statistical difference.

A total of 914 serum samples were used for quantifying the SARS-CoV-2 N protein levels. It was found that serum SARS-CoV-2 N protein level changed along with disease course (Figure 1A), and it was higher in COVID-19 patients than recovered ones and those in the control group whether patients infected with another virus (Figure 1C). Of the control group, there was no significant difference of serum SARS-CoV-2 N protein levels among subgroups (*p* > 0.05). Then 497 serum samples from 338 COVID-19 patients were classified into five groups according to days after illness onset: <7 days (weeks 1, *n* = 118), 7–14 days (weeks 2, *n* = 104), 14–21 days (weeks 3, *n*=76), 21–28 days (weeks 4, *n* = 56), and >28 days (weeks > 4, *n* = 143). The median levels of serum SARS-CoV-2 N protein were shown in Figure 1D. It was found group weeks 1 (15.02 COI) had the highest level of serum SARS-CoV-2 N protein following by group weeks 2 (6.49 COI). Positive rate of serum antigen related to time of onset (Shown in Figure 1E). In addition, we did subgroup analysis according to clinical types. One hundred and fifty-four and 83 patients were distributed into mild and severe groups, respectively. There was a significant difference in serum antigen (*p* < 0.05) between these two groups (Shown in Figure 1F).

### Serum SARS-CoV-2 Specific Antibodies Levels in Patients With COVID-19

SARS-CoV-2 specific antibodies in serum also varied across time since disease onset (Shown in Figure 1B). Both two antibodies seemed to have a delayed increase in the first week of onset, and remained at a high level for a few weeks. It conformed to the immunological characteristics of the general pathogen infection. We found that levels of antibodies in groups weeks 3 (IgM, 18.95 AU/mL; IgG, 73.73 AU/mL), weeks 4 (IgM, 17.84 AU/mL; IgG, 74.58 AU/mL), and weeks > 4 (IgM, 21.62 AU/mL; IgG, 78.01 AU/mL), were obviously higher than groups weeks 1 and weeks 2 (Figure 1G). Positive rate of serum IgM and IgG were shown in Figure 1H. The first three groups (weeks 1–3) showed significant statistical differences, but none between the last three (weeks 3, 4, and >4). Nevertheless, subgroup analysis of clinical types showed no statistical difference (*p* > 0.05) (Figure 1I).

### Diagnosis Performance of Serum SARS-CoV-2 N Protein Antigen and Specific Antibodies

**Figure 2:**
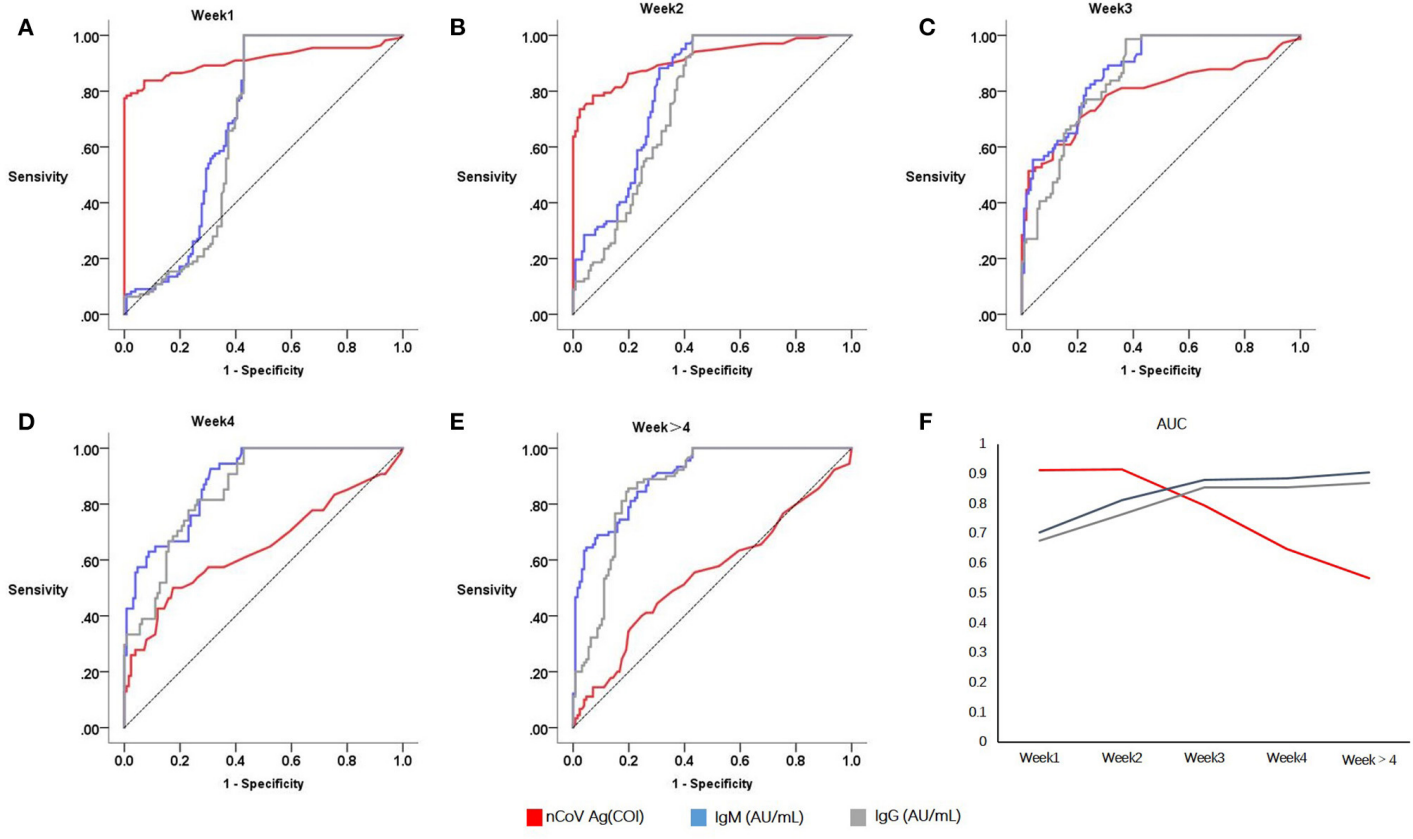
Sensitivities and specificities analysis of serological testing for COVID-19 diagnosis. Red denotes serum antigen, blue and grey denotes specific antibodies IgM and IgG, respectively. Receiver operating curves of Week1 **(A)**, Week2 **(B)**, Week3 **(C)**, Week4 **(D)**, Week > 4 **(E)**; Area under curves relate to the time of onset **(F)**.

ROC curves of serum SARS-CoV-2 N antigen for identifying COVID-19 patients were presented in Figures 2A–E. In groups weeks 1 and weeks 2, AUCs of antigen were 0.911 and 0.915 with 95% confidence interval of 0.868~0.955 and 0.876~0.954, respectively. However, in groups of patients with longer disease courses, it decreased from weeks 3 (Figure 2F), indicating poor diagnostic performance.

When it comes to antibodies, the diagnosis performance seemed to become better after acute infection. In consideration of this, serum conversion was completed after 2 weeks since symptoms onset. Both the serum specific antibody IgG and IgM had a higher AUC in group weeks 3 (IgM, 0.879; IgG, 0.855; N protein antigen, 0.794) and weeks 4 (IgM, 0.885; IgG, 0.853; N protein antigen, 0.647).

Taking diagnosis performances in different weeks of onset into account, and no significance difference between the first 2 weeks, the cut off value of serum N antigen was calculated to 1.46 based on patients within 2 weeks of onset and controls. Therefore, the test result of >1.46 COI was considered positive, tests ≤1.46 COI were regarded as negative. Of 410 control individuals, 405 were tested to be negative. Therefore, the specificity of serum SARS-CoV-2 N antigen was 98.78% (405/410). Sensitivity was calculated as 76.27% (90/118), 62.50% (65/104), in weeks 1 and 2.

### Successive Monitoring and Comparison

**Figure 3:**
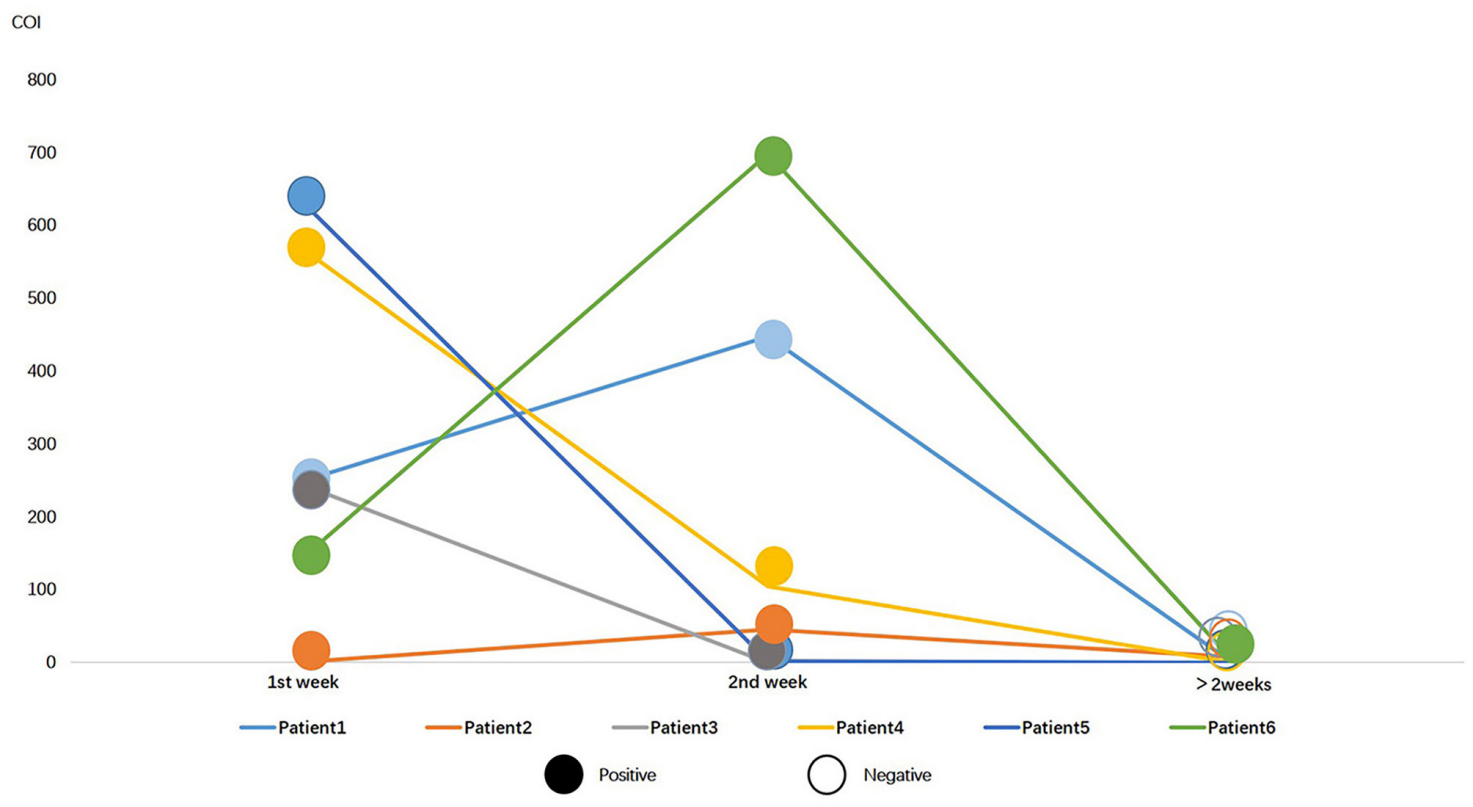
Serial data of N protein antigen and RT-PCR for 6 COVID-19 patients in different periods. Colorful lines represent patients, solid circles represent positive RT-PCR result, and hollow circles represent negative result.

Of 338 COVID-19 patients, 49 patients were tested for serum SARS-CoV-2 N antigen within and post 2 weeks after onset. Positive rate of serum antigen was significantly decreased from 77.55% (38/49) to 18.37% (9/49). Besides, six patients underwent at least three successive serological immunology analyses and nucleic acid testing. All of them had a positive result on RT-PCR in the first 2 weeks and a negative result later, except one patient, whose RT-PCT result was still positive 16 days after onset. The general trend of antigen in serum was downwards, except two which increased from the first week to the second (Figure 3).

## Discussion

In the ongoing pandemic context of COVID-19, to limit the spread of the virus and appropriately manage COVID-19 patients, it is important to use proper diagnostic testing for SARS-CoV-2. RT-PCR assay is regarded as the golden method for identifying SARS-CoV-2 infection, but performing RT-PCR assay requires special equipment and skilled laboratorians. It is costly and often time consuming, and has false negative results occasionally. Most of the commercially available rapid SARS-CoV-2 antigen test kits for swabs have low performance as frontline testing for COVID-19 diagnosis due to lower sensitivity than RT-PCR assay, particularly in respiratory tract samples (17–19). Actually, a useful viral direct detection method, serum viral antigen test, has been widely used to identify viral infectious diseases like Hepatitis B, Hepatitis C, acquired immunodeficiency syndrome, and influenza.

On the other side, it is important to optimize assays for sensitivity, robustness and automation. In the present study, a total of 10 specific antibodies against SARS-CoV-2 N protein were employed and 34 matches were compared in order to establish a highly sensitive immunoassay for the SARS-CoV-2 N antigen detection. In the second place, biosensing technologies by using magnetic beads for concentrating target proteins were applied to improve the protein sensing sensitivity. Up to now, we are not aware of any automatic method used for determining SARS-CoV-2 N or other antigen in serum. Due to high accuracy and high automaticity, chemiluminescence immunoassay for SARS-CoV-2 N antigen detection is considered appropriate for large-scale population testing as well as frontline testing for COVID-19 diagnosis.

Detection of viral antigens in the blood of COVID-19 patients has been recently described in several research studies. Ogata et al. (14) developed a single molecule array assay to quantitatively detect SARS-CoV-2 antigens in blood, finding that S1 and N antigens were detectable in 64.06% (41/64) of COVID-19 patients, similar to the result of weeks 2 in this study. Hingrat et al. (20) showed that specificity and sensitivity of the SARS-CoV-2 N antigen ELISA assay was 98.4 and 79.3%, respectively, within the first 14 days after symptoms onset. Ahava and Yuri et al. (21, 22) suggested that SARS-CoV-2 N antigen may be used as a diagnostic marker in acute COVID-19. In this research, it was observed that serum SARS-CoV-2 N antigen levels were 6.49 COI in the second week after symptoms onset, which was significantly lower than that in the first week, but higher than that in the remaining three groups. Comparative study of 49 COVID-19 patients showed a downward trend in serum N antigen when patients' conditions improved. AUCs of N antigen were 0.911 and 0.916 in the first 2 weeks after symptoms onset. It is reasonable to conclude that high levels of serum SARS-CoV-2 N antigen were generally presented in the acute phase of COVID-19, indicating that serum SARS-CoV-2 N antigen is an additionally important marker for acute phase COVID-19 diagnosis. Although of great diagnostic value, five non-COVID-19 patients serum N antigen levels were above the critical level (from 1.48 to 10.02 COI) detected by this COVID-19-CLIA, among which two were infected with flu and three were confirmed as healthy people by rechecking the clinical data. Both pathogenic nucleic acid and specific antibody IgM were negative. In analyzing the reasons, we considered that non-specific reaction might be caused by improper sample handling or hemolysis. On the other hand, it may be due to the lack of specificity of the detection method, but we could not ignore the non-specific reaction of the patients (14, 20–24).

Serum SARS-CoV-2 specific IgM and IgG were presented with low level, low prevalence, and low AUCs in COVID-19 patients in the first week after symptoms onset, then increased from the third week, which was consistent to most of the previous studies (23–26), supporting that serum SARS-CoV-2 specific IgM and IgG cannot effectively identify acute phase, convalescent, previously infected patients with COVID-19. A retrospective study conducted by Yuri et al. highlights the diagnostic value of immunoassay-based detection of serum N antigen in combination with its respective antibodies (22). Boum et al. (27) evaluated an algorithm that combined antigen rapid diagnostic test screening with antibody rapid diagnostic test screening for antigen negative, followed by PCR confirmation of IgM positive samples. However, this case identification strategy had only 34% with a slightly higher sensitivity than PCR alone, but with a specificity of 92.0% and a lower positive predictive value. Therefore, the determination of serum SARS-CoV-2 specific IgM and IgG is just a supplemental method used under the condition of a suspected patient with a negative result of RT-PCR assay (17–19, 25–27).

However, limitations existed in our work. A few outpatients had been included in this study. Due to lack of enough clinical and laboratory data, and follow up information, these patients cannot be typed according WHO recommended criteria. We were unable to analyze the actual cause of the antigen positivity in the five patients. Although the N-antigenemia was present, it was not clear whether the free viral antigens in blood have an impact on disease physiopathology. In addition, we did not find the window period of serum N antigen testing, which needed further assessment in future study.

In conclusion, accurate serum N antigen testing provides a valuable new marker for diagnosing COVID-19 and screening a large-scale population. High sensitivity of N antigen testing was found in acute phase COVID-19 patients, whereas in convalescent phase and previously infected patients, specific IgM and IgG were presented with high sensitivity, therefore, a combined test of the N antigen and specific antibody may be the optimal method for identifying SARS-CoV-2 infection.

## Data Availability Statement

The raw data supporting the conclusions of this article will be made available by the authors, without undue reservation.

## Ethics Statement

The studies involving human participants were reviewed and approved by Ethics Committee of Zhongnan Hospital, Wuhan University. Written informed consent to participate in this study was provided by the participants' legal guardian/next of kin.

## Author Contributions

YL and YP designed this study. QD and WX collected samples and data. GYe, GYa and QD performed the experiments. YP analyzed the data. QD wrote the paper with ZL and YL supervised the study. All authors agree to be accountable for the content of the work.

## Conflict of Interest

The authors declare that the research was conducted in the absence of any commercial or financial relationships that could be construed as a potential conflict of interest.

## Supplementary Material

Supplementary Data Sheet 1 Tables of candidate antibodies for SARS-CoV-2 antigen capturing and detection by CLIA.
