# Supplementary material for: High Performance of SARS-Cov-2N Protein Antigen Chemiluminescence Immunoassay as Frontline Testing for Acute Phase COVID-19 Diagnosis: A Retrospective Cohort Study
Source: Front Med (Lausanne). 2021 Jul 14;8:676560. doi: 10.3389/fmed.2021.676560 (PMC8317577; doi:10.3389/fmed.2021.676560)
Supplement: Supplementary Data Sheet 1 — Tables of candidate antibodies for SARS-CoV-2 antigen capturing and detection by CLIA. [file Data_Sheet_1.docx]

Table 1. Epitopes and types of antibodies.

| Antibody Code | Epitope | Type |
| --- | --- | --- |
| Ab01 | N’-N protein | Mono-antibody |
| Ab02 | N’-N protein | Mono-antibody |
| Ab03 | N’-N protein | Mono-antibody |
| Ab04 | C’-N protein | Mono-antibody |
| Ab05 | N’-N protein | Mono-antibody |
| Ab06 | N protein | Poly-antibody |
| Ab07 | N protein | Poly-antibody |
| Ab08 | C’-N protein | Mono-antibody |
| Ab09 | N’-N protein | Mono-antibody |
| Ab10 | C’-N protein | Mono-antibody |

Table 2. Analytical sensitivity of each antibody groups

| Groups | Capture  antibodies | Detection antibodies | Analytical sensitivity (pg/mL) |
| --- | --- | --- | --- |
| Group 1 | Ab01 | Ab02 | 2.57 |
| Group 2 |  | Ab04 | 5.80 |
| Group 3 |  | Ab06 | 8.19 |
| Group 4 |  | Ab07 | 245.24 |
| Group 5 |  | Ab08 | 21.79 |
| Group 6 |  | Ab10 | 28.99 |
| Group 7 | Ab02 | Ab04 | **0.43** |
| Group 8 |  | Ab06 | 1.31 |
| Group 9 |  | Ab07 | 123.99 |
| Group 10 |  | Ab08 | 2.43 |
| Group 11 |  | Ab10 | 7.92 |
| Group 12 | Ab03 | Ab04 | 4.85 |
| Group 13 |  | Ab06 | 3.04 |
| Group 14 |  | Ab07 | 208.55 |
| Group 15 |  | Ab08 | 31.40 |
| Group 16 |  | Ab10 | 66.97 |
| Group 17 | Ab05 | Ab04 | 1.78 |
| Group 18 |  | Ab06 | 0.88 |
| Group 19 |  | Ab07 | 290.45 |
| Group 20 |  | Ab08 | 2.61 |
| Group 21 |  | Ab10 | 3.42 |
| Group 22 | Ab06 | Ab04 | 5.33 |
| Group 23 |  | Ab07 | 913.28 |
| Group 24 |  | Ab08 | 67.17 |
| Group 25 |  | Ab10 | 50.69 |
| Group 26 | Ab07 | Ab04 | 287.65 |
| Group 27 |  | Ab06 | 805.46 |
| Group 28 |  | Ab08 | 367.58 |
| Group 29 |  | Ab10 | 532.67 |
| Group 30 | Ab09 | Ab04 | 8.08 |
| Group 31 |  | Ab06 | 10.92 |
| Group 32 |  | Ab07 | 650.35 |
| Group 33 |  | Ab08 | 71.00 |
| Group 34 |  | Ab10 | 56.42 |
